# Supplementary material for: Inhibition of TBK1/IKKε mediated RIPK1 phosphorylation sensitizes tumors to immune cell killing
Source: Cell Death Discov. 2025 Nov 28;11:551. doi: 10.1038/s41420-025-02841-x (PMC12663160; doi:10.1038/s41420-025-02841-x)
Supplement: Supplementary file 1 — Figure legends for all SI figures [file 41420_2025_2841_MOESM1_ESM.docx]

**Supplementary Figure S1.**

***Kinase activation and abundance changes of select proteins in melanoma cells sensitized to T cell killing***

**(A)** Hierarchal clustering of kinase activation signatures measured in sgCtrl or sgTRAF2 transduced D10 cells treated under the listed conditions. Sensitized sgTRAF2 cells show similar kinase activation to T cell and TNF treatment at later timepoints. Normalized Enrichment Scores (NES) calculated using PTM-SEA analysis [48]. Values represent average NES scores and * indicate significant NES with FDR < 5% in 3 biological replicates. **(B)** Protein abundance changes of dynamically regulated proteins at the phosphoproteome level following 6 hours of matched T cell co-culture using sgCtrl or sgTRAF2 transduced BLM cells with or without Birinapant treatment. Protein abundances are normalized to T0. Error bars represent standard deviation between biological replicates (n=3).

**Supplementary Figure S2**.

***Enrichment of Complex I associated proteins using TNF AP-MS and RNF31 protein levels in melanoma treatment timecourse.***

**(A)** Relative abundance of Complex I associated proteins (red dots) in sgCtrl BLM cells treated with biotin-TNF (100ng/ml) vs. cells treated with untagged TNF (100ng/ml). Horizontal line indicates P-value < 0.05, vertical lines indicate fold-change > 3, n=3 biological replicates per condition. Protein abundance values were extracted from MQ output. **(B)** Same as (A) but data performed again in a second experimental set. **(C)** Relative abundance of Complex I proteins in sensitive vs resistant tumor cell. Tumor cells were sensitized to T cell-mediated killing by knocking out RNF31 and Complex I proteins were measured by TNF-biotin AP-MS. Complex I proteins are color coded by subcomplex. Horizontal line indicates P-value < 0.05, vertical lines indicate fold-change > 4, n=3 biological replicates per condition. **(D)** Same as in (D) but tumor cells were sensitized to T cell-mediated killing through treatment with RNF31 inhibitor HOPIN8. Figure S2C,2D were created with re-used data [42]. **(E)** RNF31 protein levels in sgCtrl or sgTRAF2 transduced D10 cells treated under the listed conditions. T1= 5min for TNF treatment and 2.5 h for T cell treatment. T2= 4 h for TNF treatment and 6h for T cell treatment. Error bars represent standard deviation between biological replicates (n=3). **(F)** RNF31 protein levels in sgCtrl or sgTRAF2 transduced BLM cells treated under the listed conditions. Error bars represent standard deviation between biological replicates (n=3).

**Supplementary Figure S3**.

***Validation of TNF-AP specificity and functional KOs of TBK1 and IKKE in two human melanoma cell lines.***

**(A,B)** Functional KOs of TBK1, IKKe in two human melanoma cell lines: BLM and D10. Tumor cells were harvested and whole cell lysates were blotted against TBK1 mAb and IKKe mAb. Β-actin was used as a loading control. **(C)** WB showing different steps of TNF-AP experiment in BLM cells. Cells were treated with TNF-biotin (TNF AP condition) or untagged TNF (Neg Ctrl condition) for 10min, followed by affinity purification as described in Fig2A. Fractions of each experimental step were blotted against RIPK1 mAb.. Β-actin was used as a loading control. WCL= whole cell lysate, U.F.= Unbound fraction, EL=elution using 5% SDS. (D) Correlation between relative protein abundance of Complex I proteins in SgTBK1 vs SgCtrl D10 cells. LFQ intensity values are Log2 transformed, mean normalized, and represent the average of 2 biological replicates per condition with missing values imputed.

**Supplementary Table S2**

***Baseline expression differences between sgCtrl and sgTRAF2 for complex I proteins listed in Fig 2B.***

Baseline protein abundances (T0) in BLM sgCtrl and sgTRAF2 cells were quantified and normalized to the internal reference channel within the TMT pool. Standard deviations reflect variability across biological replicates (n = 3). Missing values for TRAF3 in sgRNA samples were imputed using the minimum value from the data matrix.

**Supplementary Table S4**.

***Target sgRNA oligonucleotide sequences CRISPR-mediated knockouts.***
