# Supplementary figures and images for: Inhibition of TBK1/IKKε mediated RIPK1 phosphorylation sensitizes tumors to immune cell killing
Source: Cell Death Discov. 2025 Nov 28;11:551. doi: 10.1038/s41420-025-02841-x (PMC12663160; doi:10.1038/s41420-025-02841-x)

Supplementary Figure 1

A

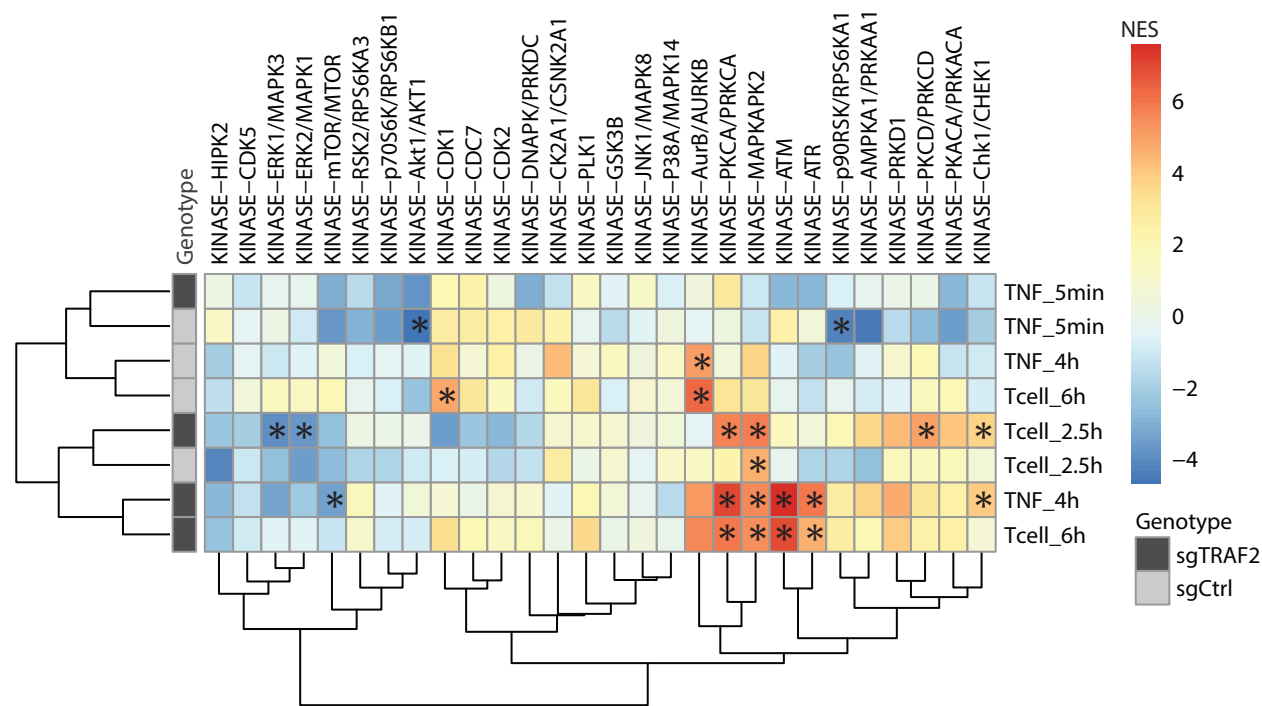

B

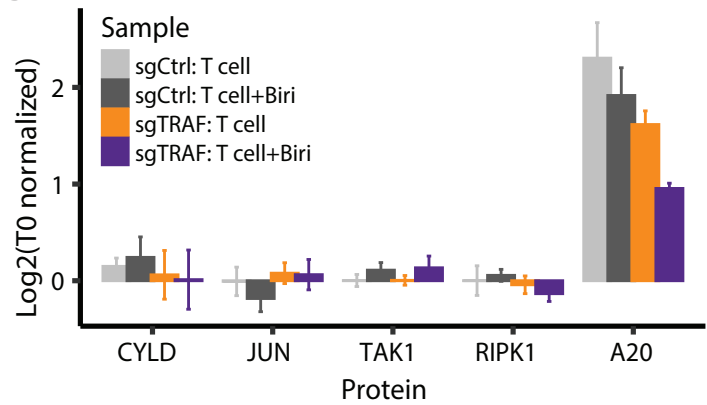

Supplementary Figure 2

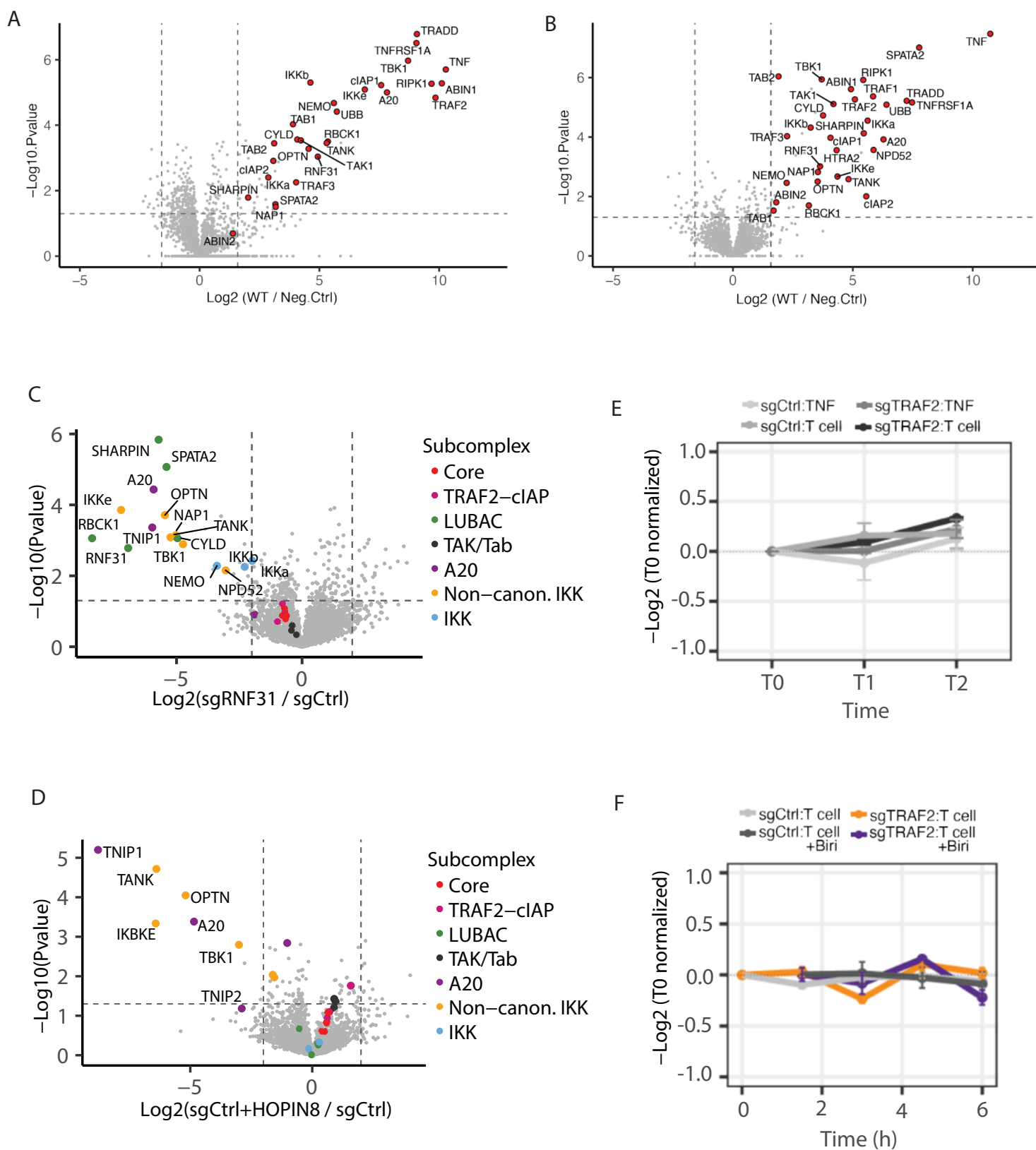

Supplementary Figure 3

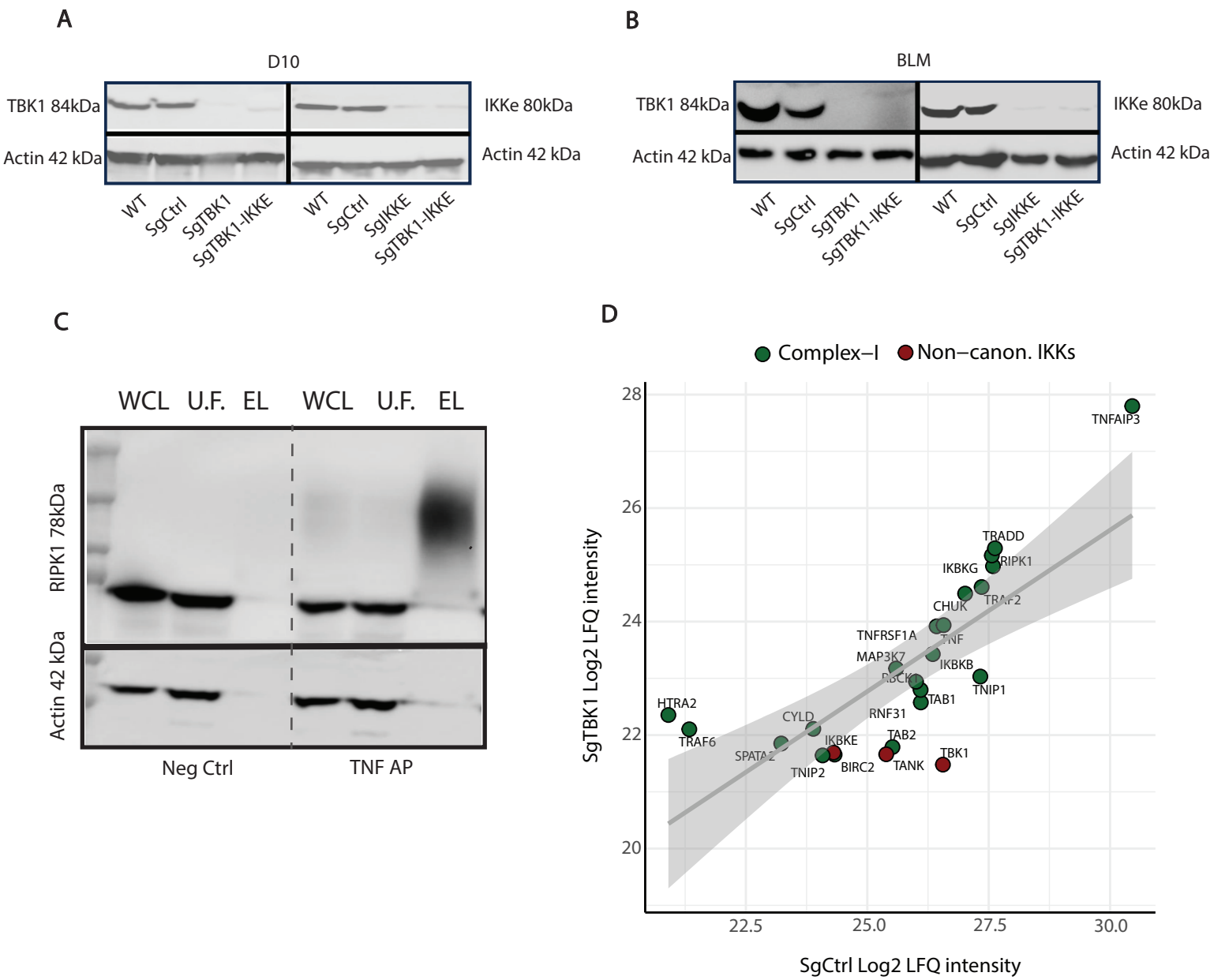

Supplement: Supplementary file 2 — All SI figures merged [file 41420_2025_2841_MOESM2_ESM.pdf]
