# Supplementary material for: Inhibition of TBK1/IKKε mediated RIPK1 phosphorylation sensitizes tumors to immune cell killing
Source: Cell Death Discov. 2025 Nov 28;11:551. doi: 10.1038/s41420-025-02841-x (PMC12663160; doi:10.1038/s41420-025-02841-x)

Supplementary Figure 3A

B-actin

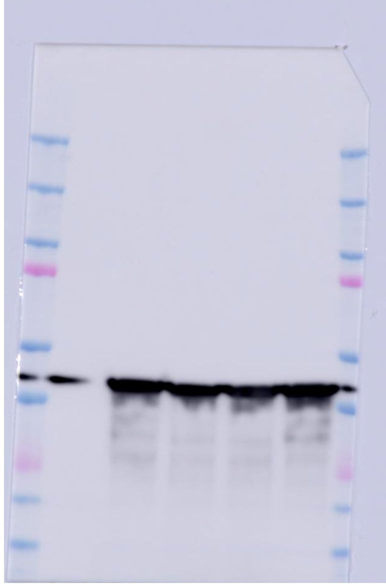

IKKe

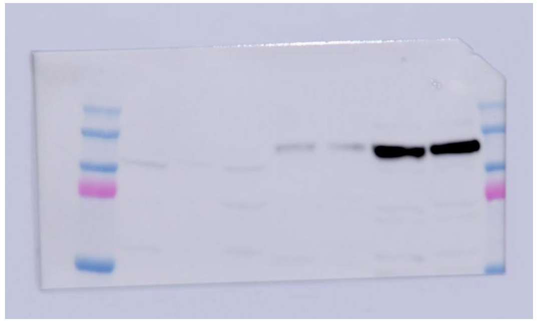

TBK1

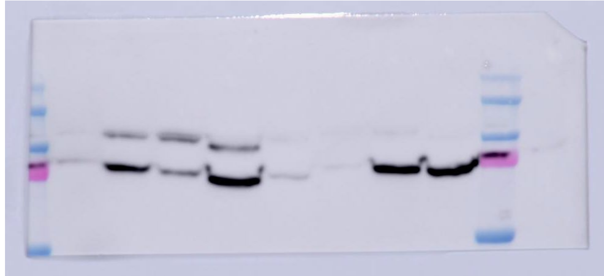

B-actin

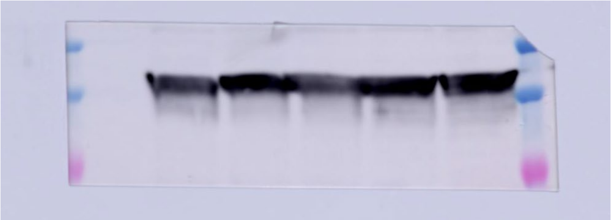

Supplementary Figure 3B

B-actin

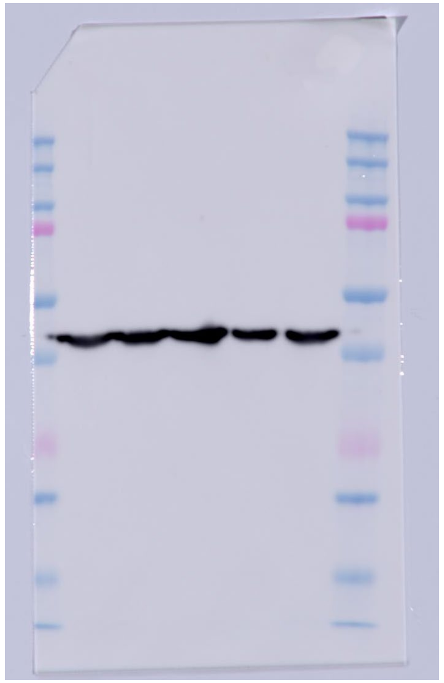

IKKe

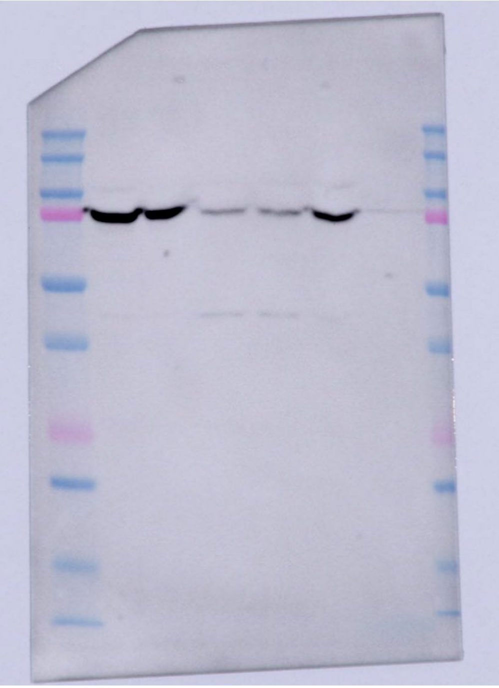

TBK1

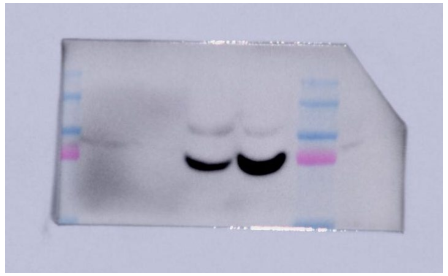

Supplementary Figure 3C

RIPK1

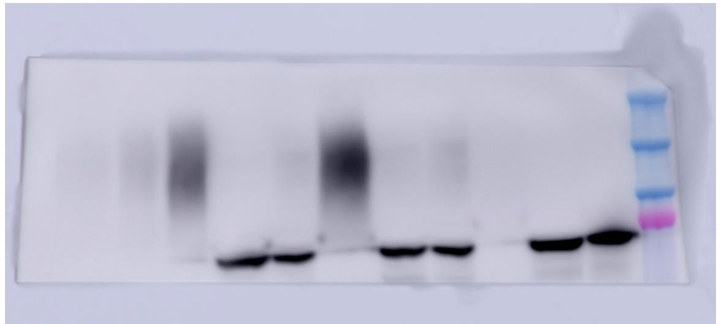

B-actin

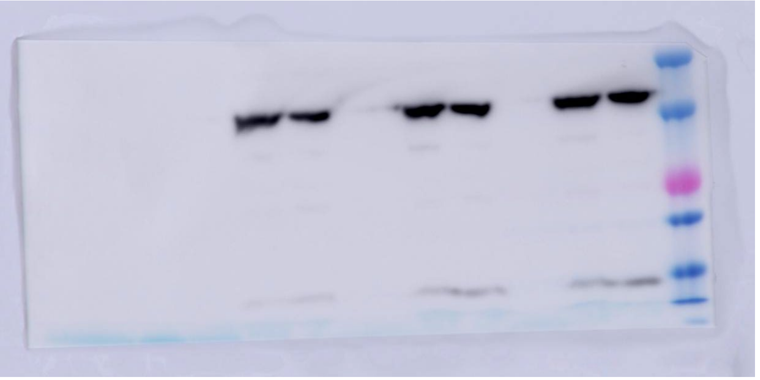

Supplement: Supplementary file 5 — All fill WB images combined [file 41420_2025_2841_MOESM5_ESM.pdf]
